# Supplementary material for: From sequence to enzyme mechanism using multi-label machine learning
Source: BMC Bioinformatics. 2014 May 19;15:150. doi: 10.1186/1471-2105-15-150 (PMC4229970; doi:10.1186/1471-2105-15-150)
Supplement: Additional file 2 — Java code of ml2db. Additional file ml2db_code.tar.gz contains the Java source code to run the multi-label machine learning experiments and save the results to database. The code’s Javadoc is included. [file 1471-2105-15-150-S2.zip › additional file 2/ml2db/ml2db/doc/mulan/evaluation/loss/package-use.html]

Uses of Package mulan.evaluation.loss


JavaScript is disabled on your browser.


- Overview
- Package
- Class
- Use
- Tree
- Deprecated
- Index
- Help

- Prev
- Next

- Frames
- No Frames

- All Classes

# Uses of Package mulan.evaluation.loss

- Packages that use mulan.evaluation.loss

  | Package | Description |
  | --- | --- |
  |  |  |
  | --- | --- |
  | mulan.classifier.neural |  |
  | mulan.evaluation.loss |  |
  | mulan.evaluation.measure |  |
- Classes in mulan.evaluation.loss used by mulan.classifier.neural

  Class and Description

  RankingLossFunction

  Interfance for ranking loss functions
- Classes in mulan.evaluation.loss used by mulan.evaluation.loss

  Class and Description

  BipartitionLossFunction

  Interfance for bipartition loss functions

  BipartitionLossFunctionBase

  Base class for bipartition loss functions

  ErrorSetSize

  Implementation of the ErrorSetSize loss function, which computes the size of
  the error set.

  MultiLabelLossFunction

  Interfance for loss functions

  RankingLossFunction

  Interfance for ranking loss functions

  RankingLossFunctionBase

  Base class for ranking loss functions
- Classes in mulan.evaluation.loss used by mulan.evaluation.measure

  Class and Description

  BipartitionLossFunction

  Interfance for bipartition loss functions

  RankingLossFunction

  Interfance for ranking loss functions

- Overview
- Package
- Class
- Use
- Tree
- Deprecated
- Index
- Help

- Prev
- Next

- Frames
- No Frames

- All Classes
